# Planting exotic relatives has increased the threat posed by *Dothistroma septosporum* to the Caledonian pine populations of Scotland

**DOI:** 10.1111/eva.12562

**Published:** 2017-11-10

**Authors:** Marta J. Piotrowska, Carolyn Riddell, Peter N. Hoebe, Richard A. Ennos

**Affiliations:** ^1^ Crop and Soil Systems Research Group Scotland's Rural College Edinburgh UK; ^2^ The Institute of Biological Chemistry, Biophysics and Bioengineering Heriot‐Watt University Edinburgh UK; ^3^ Institute of Evolutionary Biology Ashworth Laboratories University of Edinburgh Edinburgh UK; ^4^ Forest Research Northern Research Station Roslin UK

**Keywords:** *Dothistroma septosporum*, emerging disease, genetic structure, microsatellite, needle blight, pine, tree disease

## Abstract

To manage emerging forest diseases and prevent their occurrence in the future, it is essential to determine the origin(s) of the pathogens involved and identify the management practices that have ultimately caused disease problems. One such practice is the widespread planting of exotic tree species within the range of related native taxa. This can lead to emerging forest disease both by facilitating introduction of exotic pathogens and by providing susceptible hosts on which epidemics of native pathogens can develop. We used microsatellite markers to determine the origins of the pathogen *Dothistroma septosporum* responsible for the current outbreak of Dothistroma needle blight (DNB) on native Caledonian Scots pine (*Pinus sylvestris*) populations in Scotland and evaluated the role played by widespread planting of two exotic pine species in the development of the disease outbreak. We distinguished three races of *D. septosporum* in Scotland, one of low genetic diversity associated with introduced lodgepole pine (*Pinus contorta*), one of high diversity probably derived from the DNB epidemic on introduced Corsican pine (*Pinus nigra* subsp. *laricio*) in England and a third of intermediate diversity apparently endemic on Caledonian Scots pine. These races differed for both growth rate and exudate production in culture. Planting of exotic pine stands in the UK appears to have facilitated the introduction of two exotic races of *D. septosporum* into Scotland which now pose a threat to native Caledonian pines both directly and through potential hybridization and introgression with the endemic race. Our results indicate that both removal of exotic species from the vicinity of Caledonian pine populations and restriction of movement of planting material are required to minimize the impact of the current DNB outbreak. They also demonstrate that planting exotic species that are related to native species reduces rather than enhances the resilience of forests to pathogens.

## 1 INTRODUCTION

Over recent decades, a dramatic rise in the incidence of tree disease epidemics has occurred on a global scale (Stenlid, Oliva, Boberg, & Hopkins, 2011; Wingfield, Brockerhoff, Wingfield, & Slippers, 2015). Unregulated global trade in live plants, which facilitates the introduction of exotic pathogens, is largely responsible for this phenomenon (Brasier, 2008; Santini et al., 2013). However, there are a variety of other forestry practices that may be contributing significantly to our current tree disease problems (Ennos, 2015). One that deserves particular attention is the practice of planting exotic species in areas occupied by closely related native tree taxa (Burgess & Wingfield, 2017). In these situations, disease outbreaks can arise in two ways.

The first involves the transfer of endemic pathogen species or races from the native to the related exotic tree (Gilbert, Magarey, Suiter, & Webb, 2012; Gilbert & Webb, 2007). The exotic species may prove susceptible to these native pathogens due to lack of previous co‐evolution (Ennos, 2015). The natural resistance of the exotic may also be compromised because it is poorly adapted to the novel environment into which it has been planted (Karlman, Hansson, & Witzell, 1994; Read, 1968). High‐density planting in monoculture and reduced genetic diversity of the exotic host may further exacerbate disease problems. An epidemic of the native pathogen may therefore build up on the exotic plantation species. The pathogen pressure generated by this epidemic may be severe enough to produce damage on the (previously resistant) native tree species (Ennos, 2001).

The second route to epidemic disease involves the inadvertent introduction, along with the exotic tree species, of one of its co‐evolved pathogens. The native species may suffer serious damage because it has no history of co‐evolution with the introduced pathogen (Anagnostakis, 1987). Disease problems can also arise on the exotic plantation species if the novel environmental conditions that it encounters either favour the introduced pathogen directly (Gibson, 1972), or impose stress on the exotic species and increase its susceptibility to disease (Schoeneweiss, 1975, 1981). Introduced pathogens may also hybridize with closely related native pathogens to generate genotypes that are more virulent than either parent (Brasier, 2001; Brasier et al., 2004; Stukenbrock, 2016).

Well‐documented examples of disease outbreaks associated with planting of exotic relatives of native species include the white pine blister rust *Cronartium ribicola* (Lasch.) Dietr. epidemic on *Pinus strobus* L. in Europe that followed planting of this species within the native range of European five needled pines at the end of the nineteenth century (Hummer, 2000), and the epidemic of *Gremmeniella abietina* (Lagerberg) Morelet on *Pinus contorta* Douglas ex Loudon when this species was introduced into Sweden in the 1990s alongside native *Pinus sylvestris* L. (Karlman et al., 1994). More recently, the ash dieback epidemic in Europe caused by *Hymenoscyphus fraxineus* has been linked with planting of Asian *Fraxinus mandschurica* Rupr. within the native range of European ash *Fraxinus excelsior* L. (Gross, Hosoya, & Queloz, 2014).

Given the diversity of ways in which planting of exotic relatives can give rise to tree disease epidemics, detailed forensic studies of such situations are needed to establish the origin(s) of the pathogens responsible and devise appropriate control measures. If the pathogen involved is native, removal of the exotic species may be sufficient to eliminate the disease threat. However, if the pathogen is introduced, the prospects for the native species may be poor, involving death of many trees and recovery only after a prolonged period during which there is an evolution of enhanced host resistance (Gomulkiewicz & Holt, 1995). Where both exotic and native pathogens are responsible, the outcome is less predictable and will depend on the extent of genetic interactions between the pathogen sources (Brasier, 2001; Brasier et al., 2004). Here we use microsatellite markers to analyse the origin(s) of the pathogen *Dothistroma septosporum* (Dorog.) Morelet responsible for a recent outbreak of Dothistroma needle blight (DNB) on pine in Scotland (Brown, Stone, & Clayden, 2012). We highlight the role of two exotic pine species in facilitating the DNB outbreak and assess the threat that *D. septosporum* now poses to native pine populations.

Dothistroma needle blight (DNB) caused by the ascomycete *D. septosporum* is currently the most important foliar disease of pine worldwide, affecting 82 host pine species across six continents (Drenkhan et al., 2016). Needle infection by rain‐splashed conidia (Gibson, 1972) or wind‐borne ascospores (Funk & Parker, 1966) leads to defoliation, reduction in growth and, in severe cases, death of trees (Brown & Webber, 2008). *D. septosporum* is believed to be endemic on indigenous pine populations in the northern hemisphere (Drenkhan, Hantula, Vuorinen, Jankovský, & Müller, 2013; Welsh, Lewis, & Woods, 2009). From here, it has spread and caused severe damage to exotic pine plantations in the southern hemisphere (Barnes, Wingfield, Carbone, Kirisits, & Wingfield, 2014). Most recently, DNB has emerged as a problem in plantations in the northern hemisphere; in North America on *P. contorta* (Roach, Simard, & Sachs, 2015); in mainland Europe on *Pinus nigra* (Poir.) Maire (Boron, Lenart‐Boron, & Mullett, 2016; Drenkhan et al., 2016; Fabre, Ioos, Piou, & Marçais, 2012; Tomsovsky et al., 2013); and in Britain on *P. sylvestris*,* P. nigra* and *P. contorta* (Brown & Webber, 2008).

In Britain, the native host for *D. septosporum,* Scots pine *P. sylvestris,* comprises two distinct populations. Caledonian pines represent the remnants of native *P. sylvestris* populations that recolonized Scotland after the last ice age. They are confined to the Scottish Highlands and are highly fragmented, and their distribution has been reduced to less than 1% of its former area (Forestry Commission Scotland, 1998; Steven & Carlisle, 1959). Nevertheless, they retain high genetic diversity (Kinloch, Westfall, & Forrest, 1986; Wachowiak, Salmela, Ennos, Iason, & Cavers, 2011) and are of enormous conservation value because they support one of the few intact, semi‐natural forest ecosystems remaining in Britain (Mason, Hampson, & Edwards, 2004; McVean & Ratcliffe, 1962). Outside the Caledonian pinewoods, *P. sylvestris* stock derived from Forestry Commission seed orchards is used to establish plantations, and the species is extensively naturalized throughout British woodlands, representing 17% of total conifer area (Forestry Commission 2015).

Two exotic pine species closely related to Scots pine have been introduced to Britain and grown in large‐scale plantations for the last 60–100 years. Corsican pine *P. nigra* subsp. *laricio* accounts for 13% of conifer stands in England and has been successfully introduced at a small number of coastal sites in Scotland (Forestry Commission 2015). Lodgepole pine *P. contorta* of two subspecies (*contorta* and *latifolia*) is grown principally in Scotland where it makes up 10% of the conifer area (Forestry Commission 2015; Lines, 1987). Plantations of lodgepole pine often occur adjacent to or even within Caledonian pine stands.

Dothistroma needle blight (DNB) was first found in Britain in the 1950s in southern English nurseries on four exotic species: Corsican pine, lodgepole pine, *Pinus ponderosa* Douglas ex C. Lawson and *Pinus bungeana* Zucc. ex Endl. (Murray & Batko, 1962). No infection or damage to Scots pine was reported. Over the next 40 years, the presence of *D. septosporum* was recorded sporadically in southern England and southern Wales (Brown & Webber, 2008), and in the 1980s on Scots pine in northern Scotland (British Mycological Society 2014) but was not associated with significant damage. However, from 2000 onwards serious epidemics of DNB broke out in England on plantations of Corsican pine, with some infection of adjacent Scots pine. The very high level of damage led to a moratorium on plantings of Corsican pine in 2006 (Brown & Webber, 2008). Subsequently, DNB has been reported in Scotland on Corsican, lodgepole and plantation Scots pine, and on all three species in forest nurseries. Serious conservation concerns were raised in 2011 when *D. septosporum* was discovered in Caledonian pine populations where it had not previously been recorded (Brown et al., 2012).

To clarify the origins of the *D. septosporum* population in Scotland, assess the role of the exotic Corsican and lodgepole pine species in its appearance and inform management plans for its control, particularly in the Caledonian pinewoods, we initiated a detailed analysis of the genetic structure of the pathogen across its hosts within Scotland. Recent work by Mullett, Brown, Fraser, Baden, and Tubby (2017), using microsatellite marker analysis of a large sample of *D. septosporum* from across the whole of Britain, has demonstrated that individuals can be assigned to one of three major genetic groups (see Figure 2 in Mullett et al., 2017). These comprise a genetic group with low diversity, present only in Scotland and found predominantly on lodgepole pine (DAPC cluster 1 of Mullett et al., 2017) hereafter referred to as the lodgepole pine race, lodgepole pine race (LPR); a genetic group with a markedly southern distribution found largely on Corsican pine (DAPC clusters 3–7 and 10–12 of Mullett et al., 2017) designated here the southern race, SR; and a Britain wide but predominantly northern genetic grouping loosely associated with Scots pine (DAPC clusters 2, 8 and 9 of Mullett et al., 2017), named here the native pine race, NPR; LPR shows genetic similarities with samples from lodgepole pine in Canada, while SR clusters genetically with samples from northern France where it is found mainly on Corsican pine (Mullett et al., 2017).

The aim of this study was firstly to determine the involvement of the three genetic groupings of *D. septosporum* identified by Mullett et al. (2017) in the current outbreak of DNB in the native Caledonian pinewoods. We also sought to understand the degree to which the LPR, SR and NPR races are associated with different pine hosts in Scotland, and to determine the geographic distribution of these races. To do this, we designed a sampling scheme that explicitly included samples from Caledonian pinewood populations and in which we took population samples from adjacent stands of different hosts so that the effects of host species and geographic location on the frequencies of the races could be determined independently.

Four different categories of host population were recognized in the sampling; Caledonian Scots pine populations; Scots pine plantations; lodgepole pine plantations; and Corsican pine plantations. Replicate sites throughout Scotland containing adjacent stands of these different host population types were identified, and from these sites, population samples of *D. septosporum* were isolated. Clustering based on microsatellite data was used to assign individuals to races, assess the distribution of races with respect to host type within each site and to ascertain the geographic pattern of races among sites. Further samples were obtained from isolated Caledonian pinewood sites and from infected pine nurseries to measure the proportions of *D. septosporum* races present in these situations. Analysis of mating type loci and multilocus microsatellite genotypes was used to infer the reproductive systems of the three races. In addition, the races were compared in culture to establish whether they differed significantly for important phenotypic characters. We then developed a scenario, based on our results, to account for the current distribution of *D. septosporum* races in Scotland, highlighting the role played by exotic plantations of Corsican and lodgepole pine, and assessed the likely impact of *D. septosporum* on Caledonian pine populations.

## 2 MATERIALS AND METHODS

### 2.1 Sampling

We performed targeted sampling of *Dothistroma septosporum* (Dorog.) Morelet outbreaks identified in disease surveys in naturally regenerated and planted forest stands (Table 1, Figure 2). Classes of sample site and associated sampling strategies were as follows:

**Table 1:** Sampling sites, their locations and number of trees from which *Dothistroma septosporum* isolations were made

(i) Mixed plantations of Scots pines and Corsican pines					
Site	Year of collection	Total no of isolates	No of Scots pines	No of Corsican pines	Lat/Long
Culbin Forest	2015	39	20 (0:3:17)	19 (0:7:12)	57.632986 −3.6792442
Torrs Warren	2015	33	18 (0:17:1)	15 (0:12:3)	54.865287 −4.8919655
Tentsmuir	2015	30	16 (0:4:12)	14 (0:10:4)	56.413737 −2.8109709

(ii) Caledonian Scots pine sites with adjacent lodgepole pine stands					
Site	Year of collection	Total no of isolates	No of Scots pines	No of lodgepole pines	Lat/Long
Glen Einig	2014	29	23 (0:0:23)	6 (0:0:6)	57.957361 −4.7397091
Glen Garry	2014	35	20 (0:0:20)	15 (11:0:4)	57.051089 −4.9790647
Inshriach Forest	2014	32	13 (0:0:13)	19 (10:2:7)	57.097124 −3.9313490
Glen Affric	2014 2015	21	7 (0:0:7)	14 (13:0:1)	57.292428 −4.8939597
Dundreggan	2014 2015	39	29 (0:0:29)	10 (2:2:6)	57.196282 −4.7359641
Strathpeffer	2015	2	0	2 (0:1:1)	57.621310 −4.5325429

(iii) Isolated Caledonian Scots pine forests			
Site	Year of collection	No of Scots pines	Lat/Long
Glen Tanar	2015	15 (0:5:10)	57.043444 −2.8552516
Beinn Eighe	2015	23 (0:0:23)	57.629890 −5.3512581

(iv) Forest nursery sites		
Site	Year of collection	Total no of isolates
Southern Scotland	2011–2015	24 (0:23:1)
Northern Scotland	2011–2015	13 (1:3:9)
Northern England	2011–2015	3 (0:3:0)

(v) Northern American samples	
Site	No of lodgepole pines
Nass Valley, Brown Bear, British Columbia, Canada	2
Kispiox, Buckley Canyon, British Columbia, Canada	1

The number of isolates of each genetic group identified by Mullett et al. (2017) and designated lodgepole pine race (LPR), southern race (SR) and native pine race (NPR) are shown in the format (LPR:SR:NPR).

1. Mixed plantations of Scots (*P. sylvestris*) and Corsican pine (*P. nigra* subsp. *laricio*). Three sites were sampled in 2015 (Culbin Forest (*n* = 39), Torrs Warren (*n* = 33) and Tentsmuir (*n* = 30)). At each site, roughly equal numbers of isolations were made from the two host species.
2. Caledonian Scots pine sites with adjacent lodgepole pine (*P. contorta*) stands. Three sites were sampled in 2014 (Glen Einig (*n* = 29), Glen Garry (*n* = 35), Inshriach Forest (*n* = 32)) and two in both 2014 and 2015 (Glen Affric (*n* = 21), Dundreggan (*n* = 39)). At each site, we made isolations from roughly equal numbers of the two hosts. In addition, we collected two lodgepole pine isolates from Strathpeffer in 2015 (*n* = 2).
3. Caledonian Scots pine sites isolated from exotic pine plantations. Two sites were sampled in 2015, Glen Tanar (*n* = 15) and Beinn Eighe (*n* = 23).
4. Forest nursery sites with reported disease outbreaks. Samples were isolated by Forest Research (Alice Holt) during annual forest nursery DNB surveys between 2011 and 2015. Nursery samples were classified into three categories; southern Scotland (*n* = 24), northern Scotland (*n* = 13) and northern England (*n* = 3). All other information relating to the samples and their location remains confidential.

At each site, we sampled needles bearing conidiomata from 15 to 40 individuals of the relevant tree species (current year or second‐year growth needles). At Caledonian pine sites, infected needles originated mostly from naturally regenerated saplings, although in some cases mature trees were sampled. A single genotype of *D. septosporum* was obtained from each tree.

Single spore cultures were isolated following the procedure described by Mullett, Brown, and Barnes (2015), with modifications described in Piotrowska, Ennos, Riddell, and Hoebe (2016). Cultures were stored in three ways as described by Mullett and Barnes (2012); as agar cubes at 4°C, water storage at 4°C and 15% glycerol stocks at −80°C. In addition to our Scottish collection, Forest Research at Alice Holt provided a single isolate of *D. septosporum* from each of three North American populations of lodgepole pine (Nass Valley, Brown Bear (1 and 7) and Kispiox, Buckley Canyon)) (Table 1).

### 2.2 DNA extraction and genotyping

#### 2.2.1 DNA extraction

Fungal mycelium was collected from cultures on agar plates into 2‐ml cryovial tubes, freeze‐dried overnight (Alpha 1–4 LDplus, Christ, Osterode am Harz, Germany) and tissue‐lysed (Tissue Lyser LT; Qiagen, Hilden, Germany) prior to DNA extraction (~20 mg of lyophilized tissue). DNA extraction was performed using DNeasy Plant Mini Kit (Qiagen), following the manufacturer's guidelines. DNA for genotyping was re‐suspended in sterile distilled water (SDW) and stored at −20°C for further use.

#### 2.2.2 Mating type assay

Mating type variants for *D. septosporum* were determined using species‐specific primer combinations developed by Groenewald et al. (2007). The amplification reactions were carried out using GoTaq Green Master Mix (Promega, Madison, USA). Each reaction comprised 1× Promega Master Mix, 200 nM of each forward (F) and reverse (R) primers, 12.5 ng of DNA and SDW up to 25 μl. The thermocycler (GeneAmp PCR System 9700 thermocycler, Applied Biosystems, Foster City, CA, USA) conditions included initial denaturation at 94°C for 5 min, followed by 36 cycles of denaturation at 95°C for 20 s, annealing at 60°C for 30 s and extension at 72°C for 40 s, and a final extension at 72°C for 5 min. To determine mating type variants, samples were run on 1.2% agarose gels, and band sizes corresponding to mt‐1 and mt‐2 were scored manually against the Quick‐Load Purple 100 bp DNA Ladder (New England BioLabs, Ipswich, USA). Both positive and negative controls for each mating type were run on every PCR plate.

#### 2.2.3 Microsatellite scoring

To investigate the population structure of *D. septosporum*, we scored 11 microsatellite loci, using primers developed by Barnes, Cortinas, Wingfield, and Wingfield (2008). Economic fluorescence labelling (Schuelke, 2000) was used in all genotyping assays; F primers were tailed at the 5′ end with M13 universal primer and M13 primer was labelled with 6‐Carboxyfluorescein (6FAM) dye at the 5′ end. We grouped microsatellite primers into three multiplex combinations: (i) MixI: Doth_E, Doth_F, Doth_I, Doth_K, M13_FAM; (ii) MixII: Doth_J, Doth_M, Doth_DS1, Doth_DS2, M13_FAM; (iii) MixIII: Doth_G, Doth_L, Doth_O, M13_FAM. The amplification reactions were performed using Multiplex PCR Kit (Qiagen) with the following thermocycler conditions (GeneAmp PCR System 9700 thermocycler): initial denaturation at 95°C for 15 min, followed by 35 cycles of denaturation at 94°C for 30 s, annealing at 60°C for 90 s, extension at 72°C for 60 s and final extension at 60°C for 30 min. For MixI and MixII PCR, components comprised 1× Master Mix, 0.2 μM of each R and M13 primer, 0.05 μM of each F primer, 12.5 ng of DNA template and RNase free water (Qiagen) up to a final volume of 25 μl. For MixIII, the following modifications of primers’ concentrations were used: M13 at 0.2 μM, primer Doth_L at 0.2 μM of R and 0.05 μM of F, primers Doth_G and Doth_O at 0.1 μM of R and 0.025 μM of F. Genotyping reactions were run on the ABI 3730 sequencer (Applied Biosystems) at Edinburgh Genomics (UK) using size standards GS500LIZ (Life Technologies, Thermo Fisher Scientific, Wilmington, USA). Allele sizes were scored in Peak Scanner Software (v 2.0, Applied Biosystems) and binned manually for population genetic analysis.

### 2.3 Population genetic analysis of genotype data

#### 2.3.1 Genotypic clustering

To infer the number of genetic clusters within Scottish populations of *D. septosporum*, we performed analysis in R studio (v 1.0.136) using the adegenet package (v 2.0.1, Jombart, 2008; Jombart & Ahmed, 2011). The isolates were assigned to genetic groups using the multivariate discriminant analysis of principal components method (DAPC, Jombart, Devillard, & Balloux, 2010). This method of clustering was chosen because it makes no assumptions about the mating system of the organisms concerned. The optimal number of clusters was inferred using the *find.cluster* function by computing both BIC (Bayesian Information Criterion) and WSS (within sum of squares) statistics for increasing number of clusters.

#### 2.3.2 Genetic diversity and divergence among and within genetic clusters

All the input files for genetic analysis were prepared in CREATE software (v 1.37, Coombs, Letcher, & Nislow, 2008). Percentage of polymorphic loci, number of alleles, number of unique alleles possessed by each cluster, genetic diversity over all loci (*H*_*t*_) and genetic divergence (θ_*st*_) between *D. septosporum* clusters and among populations within these clusters were calculated using the FSTAT programme (v 2.9.3.2, Goudet, 1995, 2002). *H*_*t*_ was calculated according to Nei's (1987) unweighted estimator. Overall genetic differentiation between the clusters, among populations within the clusters, as well as pairwise differentiation between populations was measured using Weir and Cockerham's (1984) estimator of θ_st_. The significance of θ_st_ was tested with multiple bootstrapping over loci.

#### 2.3.3 Multilocus structure of races

For each cluster, the number of multilocus genotypes was found using the program MLGsim (Stenberg, Lundmark, & Saura, 2003). The program was also used to estimate which multilocus genotypes represented multiple times have a low probability (*p *<* *.05) of being the product of sexual reproduction. These genotypes were then treated as clonal replicates to generate a clone‐corrected data set. Allele frequencies used in the MLGsim simulation were those estimated in the complete data set (prior to removal of clonal genotypes).

Analysis of multilocus structuring of the races was conducted in three ways. In the first analysis, the program Multilocus 1.3 Beta (Agapow & Burt, 2001) was used to estimate the index of association among loci (*I*_*A*_) and mean correlation among loci *(r*_*D*_ *)*, with significance estimated using 1,000 randomizations. In the second analysis, the proportion of locus pairs showing significant association (Weir, 1996) was determined in FSTAT with allele permutations. In the third analysis, the extent of genetic differentiation between the two populations of opposite mating type within a race (θ_mt_ *)* was computed using FSTAT. If sexual reproduction is prevalent, there will be no significant genetic differentiation between populations of opposite mating type (Ennos & Hu *in prep*.). Analyses were conducted both on the original and on the clone‐corrected data sets. Additionally, the extent of genetic differentiation (θ_mt_ *)* between the clone‐corrected mt‐1 population in the SR race and the clone‐corrected mt‐1 population in the NPR race was calculated in FSTAT.

### 2.4 Analysis of growth rate and exudate production

To investigate possible genetically determined phenotypic differences between *D. septosporum* clusters identified with genetic markers and between populations within these clusters, we examined the rate of fungal colony growth and exudate production in vitro. We randomly selected five individuals from three populations within each of the three *D. septosporum* races identified above. Initial cultures were grown on Dothistroma sporulating medium (DSM) (Bradshaw, Ganley, Jones, & Dyer, 2000) at 20°C at 12‐hr dark/12‐hr light mode (Gallenkamp, INF 780C, Weiss Technik Konigswinter, Germany). Mycelial plugs of 8 mm diameter were excised from the colony and subcultured onto fresh DSM media. Samples were incubated for a further 8 weeks, in 24‐hr darkness. In total, we used six growth cabinets (3—MIR‐254 incubator; Sanyo, Osaka, Japan and 3—Gallenkamp, INF 780C), three temperatures (10°C, 17.5°C, 22.5°C) and two technical replicates for each temperature, resulting in a total number of *n* = 270 observations. The growth of isolates was measured as an increase in the colony radius (mm) from week 0 to week 8.

In culture, *D. septosporum* produces the exudate dothistromin which is known to be a virulence factor in DNB (Kabir, Ganley, & Bradshaw, 2015). Exudate production of isolates was scored at week 8 according to the degree to which it discoloured the growth medium using a 4‐point scale: no exudate = 0, low = 1, medium = 2, high = 3 (Figure S1). Statistical analysis of growth rate and exudate production was performed in Minitab v. 17 (Minitab Inc., State College, PA, USA). Data were analysed in a split plot ANOVA framework with individual incubators as plots. The analysis was used to infer the significance of the following factors: race (fixed), temperature (fixed) and their interaction; population (nested within race, random effect) and isolate (nested within isolates and populations).

## 3 RESULTS

### 3.1 Microsatellite genotyping and genotypic clustering

**Figure 1:**
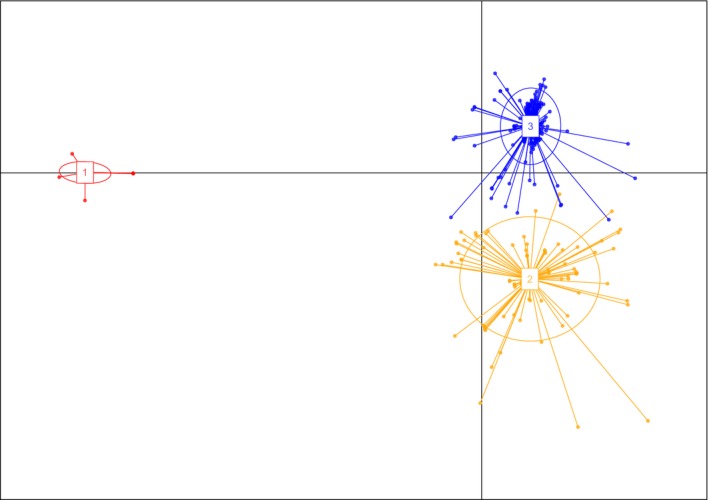
Plot of scores on first two axes from DAPC analysis (Jombart et al., 2010), based on variation at 11 microsatellite loci, for *Dothistroma septosporum* isolates from Scotland. Races LPR (red), SR (orange) and NPR (blue) are indicated

A total of *n* = 338 isolates were successfully scored for both mating type and the 11 SSR loci (Table 1). All of the SSR loci were polymorphic, but a total number of alleles per locus varied from 3 at locus O to 37 at the hypervariable M locus (Table S1). Clustering of the multilocus genotypes using DAPC analysis in the adegenet package inferred three discrete genetic clusters within the total *D. septosporum* population in Scotland (Figure 1).

### 3.2 Host and geographic distributions of genetic clusters

**Figure 2:**
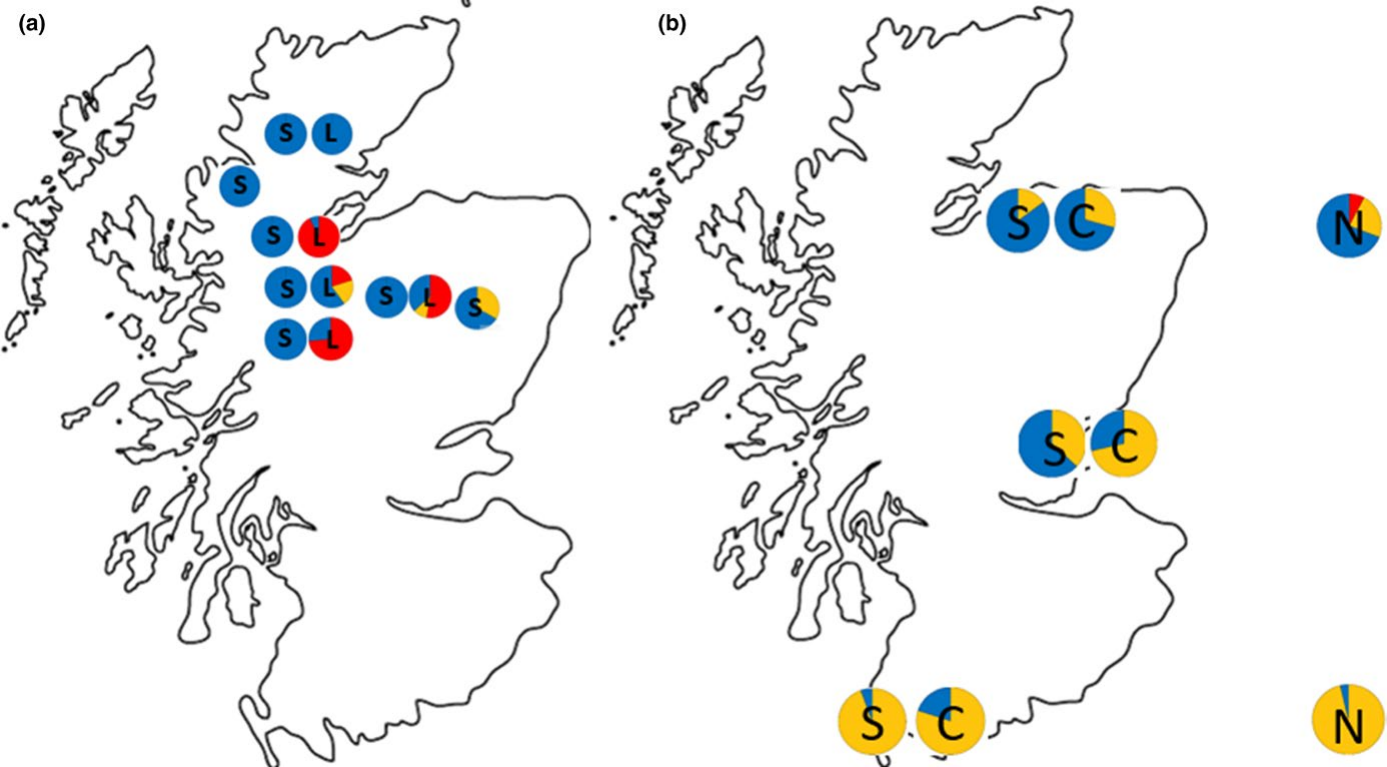
Distribution of *Dothistroma septosporum* races (LPR (red), SR (orange) and NPR (blue)); (a) on Scots (S) and lodgepole (L) pine at Caledonian pine sites, illustrating data from Table 1, sections (ii) and (iii); (b) on Scots (S) and Corsican (C) pine in mixed plantations and on pines in northern and southern Scottish nurseries (N), illustrating data from Table 1, sections (i) and (iv)

The highly genetically divergent cluster of *D. septosporum* isolates revealed by the DAPC analysis was isolated only from lodgepole pine (Table 1, Figure 2). This was true even at sites where adjacent stands of Caledonian Scots pine had been sampled and in nursery collections where isolates had also been made from both Scots and Corsican pine. This cluster will hereafter be referred to as the lodgepole pine race of *D. septosporum* (LPR). We found LPR in all sites where isolations were made from lodgepole pine with the exception of the northern Caledonian pinewood site at Glen Einig. In some locations, such as Glen Affric and Glen Garry, LPR was the predominant race on lodgepole pine, whereas in others, such as Dundreggan, it comprised a small proportion of lodgepole pine infections (Table 1, Figure 2).

In contrast to LPR, the remaining two genetic clusters distinguished by the DAPC analysis were found on all three host pine species. However, they were very distinct in their geographic distributions. The first of these clusters (*n* = 92) predominated in samples from more southern locations, and its frequency declined towards the north of Scotland (Table 1, Figure 2). In recognition of its distribution, this genetic cluster will be referred to as the southern race (SR) of *D. septosporum*. In the combined nursery samples from southern Scotland and northern England, 26 of the 27 isolates were from SR, while only 3 of 13 isolates from northern Scottish nurseries belonged to this race. In the mixed Scots and Corsican pine plantations, the frequency of SR declined from south to north and from 88% in Torrs Warren through 47% in Tentsmuir to 26% in Culbin Forest. SR was not isolated from Caledonian Scots pine trees except in the most easterly population at Glen Tanar where it was present at a frequency of 33%. In more westerly and northerly Caledonian pine sites, SR was either absent or present at a very low frequency (total 4 isolates) on lodgepole rather than Scots pine. Where it occurred at intermediate frequency in mixed plantations of Scots and Corsican pine, for example, at Tentsmuir, SR was significantly associated with Corsican pine (χ^2^ = 6.47, *df* = 1, *p *<* *.05).

The third genetic cluster recognized by the DAPC analysis (*n* = 209) was strongly associated with Caledonian Scots pine (Table 1, Figure 2). Of the 130 isolations made from Caledonian pine trees, 125 belonged to this cluster, which will henceforth be referred to as the native pine race (NPR). In addition to being present on Caledonian Scots pine, NPR was also isolated from adjacent stands of lodgepole pine and was the main race present in northern Scottish nursery samples. In mixed stands of Scots and Corsican pine, NPR predominated in the north at Culbin Forest, but its frequency declined to the south (Figure 2). Where present at intermediate frequency at Tentsmuir, it was preferentially found on Scots pine (χ^2^ = 6.47, *df* = 1, *p *<* *.05).

### 3.3 Genetic diversity and divergence among and within *Dothistroma septosporum* races

Lodgepole pine race (LPR) was the least diverse of the three *D. septosporum* races (Table 2). It was characterized by very low allelic richness *A *=* *1.55 ± 0.16, low gene diversity *H*_*t*_ = 0.041 and relatively low percentage of polymorphic loci, 55%. It had the smallest total number of alleles *n* = 17, although seven of these were private alleles distributed over six loci. Three of these alleles (E‐243, G‐195, I‐319) were shared with the three individuals sampled from North American populations (Table 1).

**Table 2:** Genetic diversity measured at 11 microsatellite loci within three *Dothistroma septosporum* races in Scotland

Race	LPR (*n* = 37)	SR (*n* = 92)	NPR (*n* = 209)
Gene diversity *H*_*t*_	0.041	0.550	0.182
% Polymorphic loci	55%	100%	91%
Total number of alleles	17	68	68
Allelic richness *A*	1.55 ± 0.16	5.50 ± 1.25	4.23 ± 1.60
Number of unique alleles	7 (over 6 loci)	21 (over 7 loci)	22 (over 4 loci)

In contrast, SR was characterized by the highest level of genetic variation (Table 2). All of its loci were polymorphic, with 68 alleles in total, 21 of which were unique and present across seven loci. SR displayed both high allelic richness *A *=* *5.50 ± 1.25 and high gene diversity *H*_*t*_ = 0.550. Allelic richness of SR was significantly higher in the population occupying plantations (*A *=* *3.75) than on Caledonian pine (*A *=* *2.64) (*p *=* *.015).

The NPR race was intermediate in genetic diversity between LPR and SR (Table 2). 91% of loci were polymorphic and total number of alleles (*n* = 68) and allelic richness *A *=* *4.23 ± 1.60 were high. However, gene diversity *H*_*t*_ = 0.182 was much lower than in SR because allelic diversity was present predominantly at a single hypervariable locus (M). NPR showed the highest number of unique alleles (*n* = 22) with 16 of these found at the hypervariable locus M (Table S1). Although mean allelic richness was greater on Caledonian pine (*A *=* *4.45) than on plantation pines (*A *=* *3.91), this difference was not significant (*p *=* *.348).

The three *D. septosporum* races were strongly genetically differentiated from each other with overall θ_st_ = 0.558, *p *<* *.01. LPR diverged the most from the other two races, showing pairwise θ_st_ = 0.8037 (*p *<* *.05) with NPR and θ_st_ = 0.5119 (*p *<* *.05) with SR. The other two races, NPR and SR, were genetically closer, with a moderate but still significant level of divergence θ_st_ = 0.3997 (*p *<* *.05).

Within the LPR race, there was no significant genetic differentiation (θ_st_ = 0.075, *p *>* *.05) among the major populations scored (Inshriach Forest, Glen Garry, Dundreggan, Glen Affric). For SR, there was low but significant genetic differentiation (θ_st_ = 0.082, *p *<* *.01) among the plantation and nursery populations (Torrs Warren, Tentsmuir, Culbin Forest, Southern Scotland nursery) (Table S2). SR also showed significant genetic differentiation between Caledonian pine and plantation populations (θ_st_ = 0.101, *p *<* *.01). In NPR, there was low but significant genetic divergence among Caledonian pine populations (θ_st_ = 0.071, *p *<* *.01) but much higher genetic differentiation among plantation populations θ_st_ = 0.318 (*p *<* *.01) (Table S3). Mean pairwise genetic differentiation between Caledonian pine populations and the northernmost Culbin Forest plantation population was low (θ_st_ = 0.040), while mean pairwise differentiation between Caledonian pine populations and the two plantation populations located further south was much higher (Tentsmuir θ_st_ = 0.147, Torrs Warren θ_st_ = 0.656) (Table S3).

### 3.4 Multilocus structure and mating type variation of races

Lodgepole pine race (LPR) comprised only five multilocus genotypes (MLGs), among which one was a clonal MLG (Table 3). All of the individuals within this race were of one mating type variant, mt‐2. There was high and significant linkage disequilibrium among loci, in both the original (*I*_*A*_ = 0.842, *r*_*D*_ = 0.176, *p *=* *.003) and clone‐corrected data sets (*I*_*A*_ = 0.576, *r*_*D*_ = 0.124, *p *=* *.015), as expected if LPR reproduces asexually.

**Table 3:** Multilocus structure of *Dothistroma septosporum* races in Scotland

Race	LPR	LPRcc^a^	SR	SRcc^a^	NPR	NPRcc^a^
mt‐1:mt‐2	0:37	0:36	45:47	26:33	7:202	7:195
Total no MLGs^b^		5		58		51
No clonal MLGs^b^		1		11		5
*I*_*A*_ c	0.842**, d	0.575*	0.663***	0.323***	2.037***	2.203***
*r*_*D*_ e	0.176**	0.124*	0.067***	0.033***	0.245***	0.261***
% pairwise l.d.^f^	0	0	46	9	38	27
*Θ*_mt_ g	–	–	0.107**	0.022*	0.378***	0.396**

^a^Clone‐corrected data set.

^b^MLG‐multilocus genotype.

^c^ *I*_*A*_—index of association among loci.

^d^Significance of deviations from expectations under purely sexual reproduction is indicated (*p <* *.05, *^*^ *p *<* *.01, *^**^ *p *<* *.001).

^e^ *r*_*D*_—mean correlation among loci.

^f^l.d.—linkage disequilibrium.

^g^ *Θ*_mt_—genetic differentiation between mating types within races.

Southern race (SR) was characterized by the highest number of MLGs *n* = 58, 11 of which were clonal MLGs (Table 3). Mating type variants, mt‐1 and mt‐2, were present in a 1:1 ratio (χ^2^ = 0.043, *df* = 1, *p *=* *.835), which suggests the possibility of sexual reproduction within SR populations. There was however significant linkage disequilibrium among loci present in the original (*I*_*A*_ = 0.663, *r*_*D*_ = 0.067, *p *<* *.001) and clone‐corrected data sets (*I*_*A*_ = 0.322, *r*_*D*_ = 0.033, *p *<* *.001). We also found a significant amount of genetic divergence between the two mating type populations (original θ_mt_ = 0.107, *p *<* *.01; clone‐corrected θ_mt_ = 0.022, *p *<* *.05), rejecting the hypothesis of random mating within SR populations. The results are compatible with SR being predominantly asexual with low levels of sexual reproduction.

A moderate number of MLGs *n* = 51 were present in the NPR population with a relatively small number of clonal MLGs (*n* = 5) (Table 3). Within NPR, multilocus genotypes were often distinguished by allelic differences at a single hypervariable locus. Linkage disequilibrium was high and significant in both the original (*I*_*A*_ = 2.037, *r*_*D*_ = 0.245, *p *<* *.001) and clone‐corrected data sets (*I*_*A*_ = 2.203, *r*_*D*_ = 0.261, *p *<* *.001). Although both mating type variants (mt‐1 and mt‐2) were present in NPR populations, the frequency of individuals bearing the mt‐1 allele was limited to only 3% of the population. Genetic differentiation was high and significant between the two mating type populations within NPR (original θ_mt_ = 0.378, *p *<* *.01; clone‐corrected θ_mt_ = 0.396, *p *<* *.01). The mating system of NPR is therefore likely to be predominantly asexual. Genotypes possessing the mt‐1 variant were noticeably more genetically variable than those possessing mt‐2 (mt‐1 *H*_*t*_ = 0.507, mt‐2 *H*_*t*_ = 0.153, *p* = .002).

### 3.5 Growth rate and exudate production of races at different temperatures

**Figure 3:**
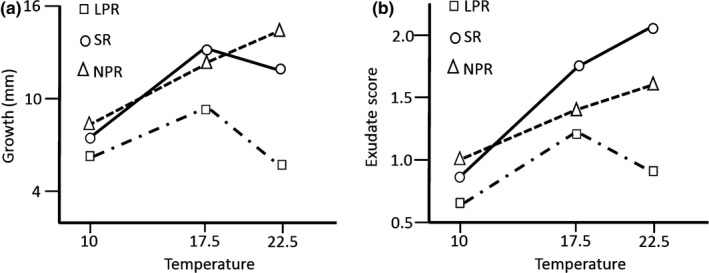
Variation in (a) growth rate and (b) exudate production at three temperatures in three races (LPR, SR and NPR) of *Dothistroma septosporum* from Scotland

We found significant differences in mycelial growth rate among the races (*F*_2,6_ = 20.40, *p *=* *.002), and significant interaction between race and temperature for this character (*F*_4,12_ = 13.31, *p *<* *.001) (Table S4) (Figure 3a). This interaction remained significant even when the LPR isolates were removed from the analysis (*F*_2,56_ = 5.93, *p *=* *.005). The three *D. septosporum* races showed different temperature optima for growth. LPR exhibited the slowest growth at all three temperatures tested, NPR was the fastest growing race at 10 and 22.5°C, while SR exhibited the fastest growth at 17.5°C.

There were also significant differences in exudate production among races (*F*_2,6_ = 11.94, *p *=* *.008) and a significant race x temperature interaction (*F*_4,12_ = 5.61, *p *=* *.009) (Table S4). LPR produced the least exudate at all three temperatures, NPR produced the most exudate at 10°C, and SR at the remaining two temperature points, 17.5 and 22.5°C (Figure 3b).

## 4 DISCUSSION

Our molecular analysis of the *D. septosporum* population in Scotland was able to assign individuals to the three major genetic groups previously recognized by Mullett et al. (2017) in their Britain wide study. In agreement with Mullett et al. (2017), we found that the first of these groups, LPR, possesses very low genetic variability and is completely asexually reproducing. In addition, our analysis of mixed sites provided no evidence of LPR on trees other than lodgepole pine, implying a high degree of host adaptation. In contrast, we found the second race SR to be highly genetically variable with the potential for sexual reproduction, again in line with the results of Mullett et al. (2017). Our results further show that within Scotland, SR is mainly southern in distribution and occurs on all three pine species, but is preferentially found on Corsican rather than Scots pine where adjacent stands of these two hosts occur, implying a modest degree of host specialization. Finally, we have shown that NPR, the third major genetic grouping distinguished by Mullett et al. (2017), is the predominant race on Caledonian pine, and preferentially infects Scots pine when present in mixture with Corsican pine. In our purely Scottish sample, NPR possesses intermediate levels of variability and reproduces largely asexually.

Besides confirming and documenting the host and geographic distributions within Scotland of the genetic groups recognized by Mullett et al. (2017), we have also shown that LPR, SR and NPR are highly significantly different both in their growth rate response to temperature and their level of production of the exudate dothistromin, implicated as a virulence factor in DNB (Kabir et al., 2015). This provides strong evidence that these groupings represent important biological, ecological and evolutionary units within *D*. *septosporum* and that it is therefore appropriate to regard them as distinct races within the species.

The very low genetic and clonal variability of LPR strongly suggests that it derives from the recent introduction of a limited number of isolates. However, despite low variability, LPR harbours seven alleles that are absent from SR and NPR, three of which were detected in the limited sample of North American isolates that were genotyped. These observations support the hypothesis of Mullett et al. (2017) that LPR has been introduced into Scotland on needle debris accompanying lodgepole pine seed imports from North America. Although seed importation is generally considered a low biosecurity risk, there are at least two other examples where it has led to transfer of important pine pathogens between North America and Europe (*Lecanosticta acicola* (Janoušek et al., 2016); *Gremmeniella abietina* (Hamelin, Lecours, & Laflamme, 1998)).

Southern race (SR) is the most genetically variable of the three races implying a large effective population size as expected in an epidemic population. Equality of mating type frequencies and low (though significant) correlation among markers together with limited differentiation across mating types suggests that SR may practise a low frequency of sexual reproduction. Therefore, some long‐distance dispersal by ascospores could occur in this race. Given the preference of SR for Corsican pine, a predominantly southern distribution, and near absence from Caledonian pine, SR is likely to have originated via recent dispersal from the epidemic of DNB on Corsican pine in England. Transfer of SR to northern plantations in Scotland via infected nursery stock is suggested firstly by the prevalence of SR on nursery material from south Scotland. In addition, the genetic composition of the SR population found on southern nursery samples is very similar to that at Tentsmuir and Culbin Forest (low genetic differentiation), a result that would be expected if infected nursery stock from southern nurseries was the source of SR at these northern sites.

The third and most enigmatic of the *D. septosporum* races, NPR, is strongly associated with Caledonian pine in our study (125 of 130 isolates derived from Caledonian pine). NPR was also isolated from lodgepole pine adjacent to Caledonian pine stands and was predominant in northern Scottish nurseries. In plantations, the frequency of NPR declined from north to south and data from Mullett et al. (2017) indicates that the equivalent “northern Scottish group” is infrequent south of the Scottish border. A plausible hypothesis to account for this host and geographic distribution is that NPR is a northern race of *D. septosporum* endemic on Caledonian pine that has recently spread to adjacent lodgepole pine stands, plantations of Corsican pine in northern Scotland and pine nurseries in the same geographic area.

Genetic support for the hypothesis that NPR represents an endemic race is equivocal. Although allelic richness is relatively high (due to hypervariability at a single locus), overall gene diversity is low. The ratio of mating types in NPR is very highly skewed, and there is a strong allelic correlation among markers and genetic differentiation between mating types, all suggesting that NPR reproduces asexually. Neither low genetic diversity nor asexual reproduction are generally considered to be attributes of endemic populations. On the other hand, NPR possesses 22 alleles that are not found in the most closely related race SR. Such race specific alleles can only have accumulated by mutation if the races have been isolated for a considerable number of generations.

To reconcile these results, we hypothesize that *D. septosporum* arrived in Scotland with pine populations colonizing from continental Europe after the last glaciation (Sinclair, Morman & Ennos, 1998). Following isolation of the Caledonian pine populations from their continental counterparts about 8 Kybp, we propose that the *D. septosporum* population went through a population bottleneck, leading to loss of genetic variation. We further propose that the population was selected for reproductive assurance under conditions at the range edge that were unfavourable for sexual reproduction, leading to the evolution of a predominantly asexual breeding system. Where environmental factors hinder outcrossing sexual reproduction, mating system transitions to uniparental reproduction are known to have taken place in a wide range of plants and animals (Avis, 2015; Holsinger, 2000) and are equally likely to have occurred in fungi (Taylor, Hann‐Sodena, Brancoa, Sylvaina, & Ellison, 2015).

If *D. septosporum* has been endemic in Caledonian pine populations, it appears curious that its presence was not noted before 2011 (Brown et al., 2012). One explanation may be that the proposed endemic race NPR, which apparently causes little damage to needles, has been overlooked, with attention being focussed on more damaging needle pathogens such as *Lophodermium seditiosum* (Minter & Millar, 1980). Another explanation may be that populations have recently increased in size due to more favourable environmental conditions for *D. septosporum*. This would be consistent with a rise in annual temperature of 0.75°C, and an increase of 23% in annual rainfall in Scotland since 1970 (Met Office, 2017). Circumstantial evidence that *D. septosporum* has had a long‐term endemic presence in the Caledonian pine populations comes from studies of geographic variation in their DNB susceptibility. Pine populations from areas of high rainfall, where conditions are most favourable to *D. septosporum*, show significantly lower susceptibility than those in low rainfall areas, the pattern expected under long‐term co‐evolution (Perry, Brown, Cavers, Cottrell, & Ennos, 2016).

Drawing together the information outlined above and that from Mullett et al. (2017), we can put forward a tentative scenario to account for the current situation in Scotland. Prior to the planting of exotic conifers in Britain, we suggest that NPR was present both as a co‐evolved endemic pathogen causing minimal damage in Caledonian pinewoods and on other Scots pine populations in Scotland. From the 1930s, Corsican pine was widely planted in England and more locally in Scotland. In the 1950s, the SR race was introduced into southern England probably from France (Mullett et al., 2017), possibly aided by transfer of diseased nursery stock. In the 1990s, SR spread to genetically susceptible high‐density Corsican pine stands in East Anglia and expanded massively to create a DNB epidemic made particularly serious by a succession of unusually wet and warm summers (Brown & Webber, 2008). High disease pressure may have facilitated limited adaptation to adjacent Scots pine, which was previously immune to attack (Murray & Batko, 1962). From these footholds, SR spread northwards through England and into Scotland in the 2000s via the stepping stones of vulnerable Corsican pine and to a lesser extent Scots pine plantations. SR has now established an outlier population in the most easterly Caledonian pine site (Glen Tanar) via natural dispersal, and in more northerly Caledonian pine sites by movement of infected lodgepole pine. The derived nature of SR in Caledonian pine sites is supported by its lower genetic diversity compared to that found on plantation pines.

Concurrent with northward movement of SR, the major Corsican pine plantation in northern Scotland (Culbin Forest) appears to have become infected by the endemic NPR race of *D. septosporum* dispersed from Caledonian pine populations present in the region, with subsequent spread to plantations further south. This direction of transfer is supported by the low differentiation between the NPR population on Caledonian pine and that at Culbin Forest, and increasing differentiation between Caledonian pine populations and plantation populations to the south. Transfer via asexual spores may have been augmented by the planting of stock infected with NPR from northern Scottish nurseries. Meanwhile, lodgepole pine plantations established in the vicinity of Caledonian pinewoods have become infected by NPR, but this has done little damage. However, the inadvertent introduction of the lodgepole pine adapted LPR race from North America has led to devastating DNB outbreaks mainly on the *latifolia* subspecies of lodgepole pine (Brown & Webber, 2008).

Our scenario implies that the planting of related exotic species has significantly altered the biodiversity threat posed by *D. septosporum* to Caledonian pine. The presence of susceptible Corsican pine throughout Britain has facilitated the introduction of race SR from continental Europe, while planted lodgepole pine has brought with it a second exotic race of *D. septosporum* LPR from North America. The two exotic races are now sympatric with race NPR which prior to their arrival was not causing any significant damage. What are the risks posed by these novel introductions?

Analysis of the outcome of natural inoculations by the SR race present at Torrs Warren showed high genetic variation for susceptibility both within and among populations of Caledonian pine (Perry, Brown et al., 2016). Thus, although SR is likely to cause some damage to Caledonian pine populations, this could be mitigated if populations are able to naturally regenerate, allowing the evolution of greater resistance to SR by natural selection (Cavers & Cottrell, 2015). Our isolation results provide no evidence that LPR has established on the Caledonian pine. Therefore, if lodgepole pine is removed from the vicinity of native Caledonian pine, the impact of LPR may be minimal. However, Mullett et al. (2017) did record rare presence of the “lodgepole population” on Scots pine, so the possibility of host switching onto Caledonian pine cannot be discounted.

Our conclusion that introduction of SR and LPR into Caledonian pine populations will have limited impact is conditional on a lack of genetic interaction among the three races of *D. septosporum* now present. In this respect, we note that introduction of SR brings with it a high frequency of isolates carrying the mt‐1 allele which is absent or at very low frequency in the other two races. If the races are sexually compatible, this opens up the possibility for hybridization between them and rapid evolution of either a new hybrid race (Brasier, 2001; Brasier et al., 2004) or introgression of important genetic attributes among the races (Paoletti, Buck, & Brasier, 2006). In either circumstance, the virulence of the races may be increased, causing more serious damage to the Caledonian pine populations (Stukenbrock, 2016).

Tentative evidence for hybridization between NPR and SR comes from an analysis of the small number of NPR isolates from Caledonian pine that carry the mt‐1 allele. Genetic differentiation of these isolates from mt‐2 isolates of the NPR mating type is much larger (θ_st_ = 0.369, *p* < .01) than from mt‐1 isolates in the SR race (θ_st_ = 0.048, n.s.). A possible explanation is that the mt‐1 mating types within NPR are actually hybrids between the SR and NPR races. Analysis of the genomic constitution of these isolates, now underway, will provide further evidence for or against this hypothesis (Stukenbrock, 2016).

Possible steps to reduce the spread of SR into Caledonian pine include a moratorium on movement of nursery stock of either Scots or lodgepole pine into Caledonian pinewood sites, and removal of plantations of either Scots, lodgepole or Corsican pine from areas within the range of sexual spore dispersal. If the SR race nevertheless becomes established within Caledonian pine populations, control of DNB would be best achieved by promoting management of the pinewoods for natural regeneration (Cavers & Cottrell, 2015) to maximize the opportunity for greater resistance to evolve (Perry, Wachowiak et al., 2016; Perry, Brown et al., 2016).

Overall, our results demonstrate the power of adopting a forensic forest pathology approach in which population genetic analysis of molecular markers is used to unravel the origins and subsequent evolution of emerging forest pathogens. The approach has already provided previously inaccessible information on the introduction pathways and subsequent behaviour of forest pathogens in genera such as *Heterobasidion* (Garbelotto, Guglielmo, Mascheretti, Croucher, & Gonthier, 2013), *Cryphonectria* (Dutech, Fabreguettes, Capdevielle, & Robin, 2010) and *Hymenoschyphus* (Gross et al., 2014). Crucially, this approach can identify previously cryptic but evolutionarily important units within recognized morphological taxa which may possess very different ecological attributes (Perez et al., 2012). Understanding these ecological differences may be key to explaining the epidemiology of the associated disease.

From a wider forest policy viewpoint, our analysis of DNB in Scotland provides a clear illustration of the dangers of establishing plantations of exotic tree taxa that are related to and share pathogens with native tree species (Burgess & Wingfield, 2017; Gilbert & Webb, 2007; Gilbert et al., 2012). Establishment of exotic Corsican and lodgepole pine has led to two separate and economically damaging epidemics of DNB on exotic plantations, caused by two new races of DNB that accompanied their exotic hosts. In addition, the presence of these exotic pathogen races has increased the biosecurity threat to the iconic Caledonian pine populations. Our results are highly relevant to the recent debate over the merits of introducing exotic species to increase the diversity and resilience of ecosystems (Schlaepfer, Sax, & Olden, 2011; Vitule, Freire, Vazquez, Nunez, & Simberloff, 2012). Our overall conclusion, based on the outcome of a large scale though unplanned historical experiment, is that planting exotic trees related to native species is likely to decrease rather than increase the resilience of forest ecosystems to disease.

## AUTHOR CONTRIBUTIONS

MJP, CR, PNH and RAE were all involved in the design and performance of the research together with data collection, analysis and interpretation. MJP and RAE wrote the manuscript with significant input from CR and PNH.

## DATA ACCESSIBILITY

Data available from the Dryad Digital Repository: https://doi.org/10.5061/dryad.7p5s7.

## References

[eva12562-bib-0001] Agapow, P. M. , & Burt, A. (2001). Indices of multilocus linkage disequilibrium. Molecular Ecology Notes, 1, 101–102. https://doi.org/10.1046/j.1471-8278.2000.00014.x

[eva12562-bib-0002] Anagnostakis, S. L. (1987). Chestnut blight: The classical problem of an introduced pathogen. Mycologia, 79, 23–37. https://doi.org/10.2307/3807741

[eva12562-bib-0003] Avis, J. C. (2015). Evolutionary perspectives on clonal reproduction in vertebrate animals. Proceedings of the National Academy of Sciences, 112, 8867–8873. https://doi.org/10.1073/pnas.1501820112 10.1073/pnas.1501820112 PMC4517198 26195735

[eva12562-bib-0004] Barnes, I. , Cortinas, M. N. , Wingfield, M. J. , & Wingfield, B. D. (2008). Microsatellite markers for the red band needle blight pathogen, *Dothistroma septosporum* . Molecular Ecology Resources, 8, 1026–1029. https://doi.org/10.1111/men.2008.8.issue-5 21585961 10.1111/j.1755-0998.2008.02142.x

[eva12562-bib-0005] Barnes, I. , Wingfield, M. J. , Carbone, I. , Kirisits, T. , & Wingfield, B. (2014). Population structure and diversity of an invasive pine needle pathogen reflects anthropogenic activity. Ecology and Evolution, 4, 3642–3661. https://doi.org/10.1002/ece3.2014.4.issue-18 25478155 10.1002/ece3.1200 PMC4224538

[eva12562-bib-0006] Boron, P. , Lenart‐Boron, A. , & Mullett, M. (2016). The distribution of *Dothistroma septosporum* and its mating types in Poland. Forest Pathology, 46, 489–496. https://doi.org/10.1111/efp.12262

[eva12562-bib-0007] Bradshaw, R. E. , Ganley, R. J. , Jones, W. T. , & Dyer, P. S. (2000). High levels of dothistromin toxin produced by the forest pathogen *Dothistroma pini* . Mycological Research, 104, 325–332. https://doi.org/10.1017/S0953756299001367

[eva12562-bib-0008] Brasier, C. M. (2001). Rapid evolution of introduced plant pathogens via interspecific hybridization: Hybridization is leading to rapid evolution of Dutch elm disease and other fungal plant pathogens. BioScience, 51, 123–133. https://doi.org/10.1641/0006-3568(2001)051[0123:REOIPP]2.0.CO;2

[eva12562-bib-0009] Brasier, C. M. (2008). The biosecurity threat to the UK and global environment from international trade in plants. Plant Pathology, 57, 792–808. https://doi.org/10.1111/ppa.2008.57.issue-5

[eva12562-bib-0010] Brasier, C. M. , Kirk, S. A. , Declan, J. , Cooke, D. E. L. , Jung, T. , & Man In't Velda, W.A. (2004). *Phytophthora alni* sp. nov. and its variants: Designation of emerging heteroploid hybrid pathogens spreading on *Alnus* trees. Mycological Research, 108, 1172–1184. https://doi.org/10.1017/S0953756204001005 15535068 10.1017/s0953756204001005

[eva12562-bib-0011] British Mycological Society (2014) The Fungal Records Database of Britain and Ireland. http://www.fieldmycology.net/FRDBI/FRDBIrecord.asp?intGBNum-7910

[eva12562-bib-0012] Brown, A. V. , Stone, C. , & Clayden, H. (2012). Dothistroma needle blight: GB strategy. Edinburgh, UK: Forestry Commission.

[eva12562-bib-0013] Brown, A. , Webber, J. . (2008) Red band needle blight of conifers in Britain. Forestry Commission Research Note, June 2008. Online. Available from: http://www.forestry.gov.uk/pdf/FCRN002.pdf/FILE/FCRN002.pdf. [Accessed: 13/04/2017].

[eva12562-bib-0014] Burgess, T. I. , & Wingfield, M. J. (2017). Pathogens on the move: A 100‐year global experiment with planted Eucalypts. BioScience, 67, 14–25. https://doi.org/10.1093/biosci/biw146

[eva12562-bib-0015] Cavers, S. , & Cottrell, J. E. (2015). The basis of resilience in forest tree species and its use in adaptive forest management in Britain. Forestry, 88, 13–26. https://doi.org/10.1093/forestry/cpu027

[eva12562-bib-0016] Coombs, J. , Letcher, B. , & Nislow, K. (2008). CREATE: A software to create input files from diploid genotypic data for 52 genetic software programs. Molecular Ecology Resources, 8, 578–580. https://doi.org/10.1111/j.1471-8286.2007.02036.x 21585837 10.1111/j.1471-8286.2007.02036.x

[eva12562-bib-0017] Drenkhan, R. , Hantula, J. , Vuorinen, M. , Jankovský, L. , & Müller, M. M. (2013). Genetic diversity of *Dothistroma septosporum* in Estonia, Finland and Czech Republic. European Journal of Plant Pathology, 136, 71–85. https://doi.org/10.1007/s10658-012-0139-6

[eva12562-bib-0018] Drenkhan, R. , Tomesova‐Haataja, V. , Fraser, S. , Bradshaw, R. E. , Vahalík, P. , Mullett, M. S. , … Barnes, I. (2016). Global geographic distribution and host range of Dothistroma species: A comprehensive review. Forest Pathology, 46, 408–442. https://doi.org/10.1111/efp.12290

[eva12562-bib-0019] Dutech, C. , Fabreguettes, O. , Capdevielle, X. , & Robin, C. (2010). Multiple introductions of divergent genetic lineages in an invasive fungal pathogen, Cryphonectria parasitica, in France. Heredity, 105, 220–228. https://doi.org/10.1038/hdy.2009.164 19997121 10.1038/hdy.2009.164

[eva12562-bib-0020] Ennos, R. A. (2001). The introduction of lodgepole pine as a major forest crop in Sweden: Implications for host–pathogen evolution. Forest Ecology and Management, 141, 85–96. https://doi.org/10.1016/S0378-1127(00)00491-6

[eva12562-bib-0021] Ennos, R. A. (2015). Resilience of forests to pathogens: An evolutionary ecology perspective. Forestry, 88, 41–52. https://doi.org/10.1093/forestry/cpu048

[eva12562-bib-0022] Fabre, B. , Ioos, R. , Piou, D. , & Marçais, B. (2012). Is the emergence of Dothistroma needle blight of pine in France caused by the cryptic species *Dothistroma pini*? Phytopathology, 102, 47–54. https://doi.org/10.1094/PHYTO-02-11-0036 22165983 10.1094/PHYTO-02-11-0036

[eva12562-bib-0023] Forestry Commission (2015) Forestry Statistics 2015‐National Forest Inventory [WWW Document].URL. http://www.forestry.gov.uk/website/forstats2015.nsf/LUContents/4140C187A4F725E6802573610030EFB8 (accessed 9.03.17).

[eva12562-bib-0024] Forestry Commission Scotland (1998). Caledonian pinewood inventory. Edinburgh, UK: Forestry Commission.

[eva12562-bib-0025] Funk, A. , & Parker, A. K. (1966). *Scirrhia pini* n. sp., the perfect state of *Dothistroma pini* Hulbary. Canadian Journal of Botany, 44, 1171–1176. https://doi.org/10.1139/b66-128

[eva12562-bib-0026] Garbelotto, M. , Guglielmo, F. , Mascheretti, S. , Croucher, P. J. P. , & Gonthier, P. (2013). Population genetic analyses provide insights on the introduction pathway and spread patterns of the North American forest pathogen *Heterobasidion irregulare* in Italy. Molecular Ecology, 22, 4855–4869. https://doi.org/10.1111/mec.12452 24033583 10.1111/mec.12452

[eva12562-bib-0027] Gibson, I. A. S. (1972). Dothistroma needle blight of *Pinus radiata* . Annual Review of Phytopathology, 10, 51–72. https://doi.org/10.1146/annurev.py.10.090172.000411

[eva12562-bib-0028] Gilbert, G. S. , Magarey, R. , Suiter, K. , & Webb, C. O. (2012). Evolutionary tools for phytosanitary risk analysis: Phylogenetic signal as a predictor of host range of plant pests and pathogens. Evolutionary Applications, 5, 869–878. https://doi.org/10.1111/j.1752-4571.2012.00265.x 23346231 10.1111/j.1752-4571.2012.00265.x PMC3552404

[eva12562-bib-0029] Gilbert, G. S. , & Webb, C. O. (2007). Phylogenetic signal in plant pathogen–host range. Proceedings of the National Academy of Sciences, 104, 4979–4983. https://doi.org/10.1073/pnas.0607968104 10.1073/pnas.0607968104 PMC1829250 17360396

[eva12562-bib-0030] Gomulkiewicz, R. , & Holt, R. D. (1995). When does evolution by natural selection prevent extinction? Evolution, 49, 201–207. https://doi.org/10.1111/evo.1995.49.issue-1 28593677 10.1111/j.1558-5646.1995.tb05971.x

[eva12562-bib-0031] Goudet, J. (1995). FSTAT (version 1.2): A computer program to calculate F‐statistics. Journal of Heredity, 86, 485–486. https://doi.org/10.1093/oxfordjournals.jhered.a111627

[eva12562-bib-0032] Goudet, J . (2002) FSTAT, a program to estimate and test gene diversities and fixation indices (version 2.9.3.2). Updated from Goudet (1995). [WWW document] URL http://www2.unil.ch/popgen/softwares/fstat.htm. [accessed 22 February 2017].

[eva12562-bib-0033] Groenewald, M. , Barnes, I. , Bradshaw, R. E. , Brown, A.V. , Dale, A. , Groenewald, J.Z. , … Crous, P.W. (2007). Characterization and distribution of mating type genes in the Dothistroma needle blight pathogens. Mycology, 97, 825–834. doi: 10.1094/PHYTO-97-7-0825. 10.1094/PHYTO-97-7-0825 18943931

[eva12562-bib-0034] Gross, A. , Hosoya, T. , & Queloz, V. (2014). Population structure of the invasive forest pathogen *Hymenoscyphus pseudoalbidus* Molecular Ecology, 23, 2943–2960. https://doi.org/10.1111/mec.12792 24819666 10.1111/mec.12792

[eva12562-bib-0035] Hamelin, R. C. , Lecours, N. , & Laflamme, G. (1998). Molecular evidence of distinct introductions of the European race of *Gremmeniella abietina* into North America. Phytopathology, 88, 582–588. https://doi.org/10.1094/PHYTO.1998.88.6.582 18944913 10.1094/PHYTO.1998.88.6.582

[eva12562-bib-0036] Holsinger, K. E. (2000). Reproductive systems and evolution in vascular plants. Proceedings of the National Academy of Sciences, 97, 7037–7042. https://doi.org/10.1073/pnas.97.13.7037 10.1073/pnas.97.13.7037 PMC34381 10860968

[eva12562-bib-0037] Hummer, K. E. (2000). History of the origin and dispersal of white pine blister rust. Hortechnology, 10, 515–517.

[eva12562-bib-0038] Janoušek, J. , Wingfield, M. J. , Monsivais, J. G. , Jankovský, L. , Stauffer, C. , Konečný, A. , & Barnes, I. (2016). Genetic analyses suggest separate introductions of the pine pathogen *Lecanosticta acicola* into Europe. Phytopathology, 106, 1413–1425. https://doi.org/10.1094/PHYTO-10-15-0271-R 26714104 10.1094/PHYTO-10-15-0271-R

[eva12562-bib-0039] Jombart, T. (2008). adegenet: A R package for the multivariate analysis of genetic markers. Bioinformatics, 24, 1403–1405. https://doi.org/10.1093/bioinformatics/btn129 18397895 10.1093/bioinformatics/btn129

[eva12562-bib-0040] Jombart, T. , & Ahmed, I. (2011). adegenet 1.3‐1: New tools for the analysis of genome‐wide SNP data. Bioinformatics, 27, 3070–3071. https://doi.org/10.1093/bioinformatics/btr521 21926124 10.1093/bioinformatics/btr521 PMC3198581

[eva12562-bib-0041] Jombart, T. , Devillard, S. , & Balloux, F. (2010). Discriminant analysis of principal components: A new method for the analysis of genetically structured populations. BMC Genetics, 11, 94 https://doi.org/10.1186/1471-2156-11-94 20950446 10.1186/1471-2156-11-94 PMC2973851

[eva12562-bib-0042] Kabir, M. S. , Ganley, R. J. , & Bradshaw, R. E. (2015). Dothistromin toxin is a virulence factor in dothistroma needle blight of pines. Plant Pathology, 64, 225–234. https://doi.org/10.1111/ppa.2015.64.issue-1

[eva12562-bib-0043] Karlman, M. , Hansson, P. , & Witzell, J. (1994). Scleroderris canker on lodgepole pine introduced in northern Sweden. Canadian Journal of Forest Research, 24, 1948–1959. https://doi.org/10.1139/x94-250

[eva12562-bib-0044] Kinloch, B. B. , Westfall, R. D. , & Forrest, G. I. (1986). Caledonian Scots pine; origins and genetic structure. New Phytologist, 104, 703–729. https://doi.org/10.1111/nph.1986.104.issue-4 10.1111/j.1469-8137.1986.tb00671.x 33873854

[eva12562-bib-0045] Lines, R . (1987) Choice of seed origins for the main forest species in Britain. Forestry Commission Bulletin 66. London, UK: HMSO.

[eva12562-bib-0046] Mason, W. L. , Hampson, A. , & Edwards, C. (2004). Managing the pinewoods of Scotland. Edinburgh, UK: Forestry Commission.

[eva12562-bib-0047] McVean, D. N. , & Ratcliffe, D. A. (1962). Plant communities of the Scottish Highlands. A study of Scottish mountain, moorland and forest vegetation. Monograph on Nature Conservation, 1, 1–458.

[eva12562-bib-0048] Met Office (2017). http://www.metoffice.gov.uk/climate/uk/summaries/actualmonthly [accessed 3 August 2017].

[eva12562-bib-0049] Minter, D.W. , Millar, C.S. (1980). Ecology and biology of three Lophodermium species on secondary needles of *Pinus sylvestris* . European Journal of Forest Pathology, 10, 169–181. https://doi.org/10.1111/efp.1980.10.issue-2-3

[eva12562-bib-0050] Mullett, M.S. , Barnes, I. . (2012) Dothistroma isolation and molecular identification methods. COST ACTION FP1102. Determining Invasiveness and Risk of Dothistroma. [WWW document]URL http://www.forestry.gov.uk/pdf/DIAROD_052012_Isolation_and_indentification.pdf/FILE/DIAROD_052012_Isolation_and_indentification.pdf. [accessed 21 February 2017].

[eva12562-bib-0051] Mullett, M. S. , Brown, A. V. , & Barnes, I. (2015). Population structure and reproductive mode of *Dothistroma septosporum* in the Brittany peninsula of France. European Journal of Plant Pathology, 143, 261–275. https://doi.org/10.1007/s10658-015-0678-8

[eva12562-bib-0052] Mullett, M. , Brown, A. V. , Fraser, S. , Baden, R. , & Tubby, K. V. (2017). Insights into the pathways of spread and potential origins of *Dothistroma septosporum* in Britain. Fungal Ecology, 26, 85–98. https://doi.org/10.1016/j.funeco.2017.01.002

[eva12562-bib-0053] Murray, J. S. , & Batko, S. (1962). *Dothistroma pini* Hulbary: A new disease on pine in Britain. Forestry, 34, 57–65. https://doi.org/10.1093/forestry/35.1.57

[eva12562-bib-0054] Nei, M. (1987). Molecular Evolutionary Genetics. New York, USA: Columbia University Press.

[eva12562-bib-0055] Paoletti, M. , Buck, K. W. , & Brasier, C. M. (2006). Selective acquisition of novel mating type and vegetative incompatibility genes via interspecies gene transfer in the globally invading eukaryote *Ophiostoma novo‐ulmi* . Molecular Ecology, 15, 249–262. 16367844 10.1111/j.1365-294X.2005.02728.x

[eva12562-bib-0056] Perez, G. , Slippers, B. , Wingfield, M. J. , Wingfield, B. D. , Carnegie, A. J. , & Burgess, T. I. (2012). Cryptic species, native populations and biological invasions by a eucalypt forest pathogen. Molecular Ecology, 21, 4452–4471. https://doi.org/10.1111/j.1365-294X.2012.05714.x 22882273 10.1111/j.1365-294X.2012.05714.x

[eva12562-bib-0057] Perry, A. , Brown, A. V. , Cavers, S. , Cottrell, J. E. , & Ennos, R. A. (2016). Has Scots pine (*Pinus sylvestris*) co‐evolved with *Dothistroma septosporum* in Scotland? Evidence for spatial heterogeneity in the susceptibility of native provenances. Evolutionary Applications, 9, 982–993. https://doi.org/10.1111/eva.2016.9.issue-8 27606006 10.1111/eva.12395 PMC4999528

[eva12562-bib-0058] Perry, A. , Wachowiak, W. , Brown, A. V. , Ennos, R. A. , Cottrell, J. E. , & Cavers, S. (2016). Substantial heritable variation for susceptibility to *Dothistroma septosporum* within populations of native British Scots pine (*Pinus sylvestris*). Plant Pathology, 65, 987–996. https://doi.org/10.1111/ppa.2016.65.issue-6 27587900 10.1111/ppa.12528 PMC4984854

[eva12562-bib-0059] Piotrowska, M. J. , Ennos, R. A. , Riddell, C. , & Hoebe, P. N. (2016). Fungicide sensitivity of *Dothistroma septosporum* isolates in the UK. Forest Pathology, 47, e12314.

[eva12562-bib-0060] Read, D. J. (1968). Some aspects of the relationship between shade and fungal pathogenicity in an epidemic disease of pine. New Phytologist, 67, 39–48. https://doi.org/10.1111/nph.1968.67.issue-1

[eva12562-bib-0061] Roach, W. J. , Simard, S. W. , & Sachs, D. L. (2015). Evidence against planting lodgepole pine monocultures in the cedar–hemlock forests of southeastern British Columbia. Forestry, 88, 345–358. https://doi.org/10.1093/forestry/cpv005

[eva12562-bib-0062] Santini, A. , Ghelardini, L. , De Pace, C. , Desprez‐Loustau, M.L. , Capretti, P. , Chandelier, A. , … Stenlid, J. (2013). Biogeographical patterns and determinants of invasion by forest pathogens in Europe. New Phytologist, 197, 238–250. https://doi.org/10.1111/j.1469-8137.2012.04364.x 23057437 10.1111/j.1469-8137.2012.04364.x

[eva12562-bib-0063] Schlaepfer, M. A. , Sax, D. F. , & Olden, J. D. (2011). The potential conservation value of non‐native species. Conservation Biology, 25, 428–437. https://doi.org/10.1111/j.1523-1739.2010.01646.x 21342267 10.1111/j.1523-1739.2010.01646.x

[eva12562-bib-0064] Schoeneweiss, D. F. (1975). Predisposition, stress and plant disease. Annual Review of Phytopathology, 13, 193–211. https://doi.org/10.1146/annurev.py.13.090175.001205

[eva12562-bib-0065] Schoeneweiss, D. F. (1981). The role of environmental stress in disease of woody plants. Plant Disease, 65, 308–314. https://doi.org/10.1094/PD-65-308

[eva12562-bib-0066] Schuelke, M. (2000). An economic method for the fluorescent labelling of PCR fragments. Nature Biotechnology, 18, 233–234. https://doi.org/10.1038/72708 10.1038/72708 10657137

[eva12562-bib-0501] Sinclair, W. T. , Morman, J. D. , & Ennos, R. A. (1998). Multiple origins for Scots pine (Pinus sylvestris L.) in Scotland: Eevidence from mitochondrial DNA variation. Heredity, 80, 233–240. http://doi.org/10.1046/j.1365-2540.1998.00287.x

[eva12562-bib-0067] Stenberg, P. , Lundmark, M. , & Saura, A. (2003). MLGsim: A program for detecting clones using a simulation approach. Molecular Ecology Resources, 3, 329–331. https://doi.org/10.1046/j.1471-8286.2003.00408.x

[eva12562-bib-0068] Stenlid, J. , Oliva, J. , Boberg, J. B. , & Hopkins, A. J. M. (2011). Emerging diseases in European forest ecosystems and responses in society. Forests, 2, 486–504. https://doi.org/10.3390/f2020486

[eva12562-bib-0069] Steven, H. M. , & Carlisle, A. (1959). The native pinewoods of Scotland. Edinburgh, UK: Oliver and Boyd.

[eva12562-bib-0070] Stukenbrock, E. H. (2016). The role of hybridization in the evolution and emergence of new fungal plant pathogens. Phytopathology, 106, 104–112. https://doi.org/10.1094/PHYTO-08-15-0184-RVW 26824768 10.1094/PHYTO-08-15-0184-RVW

[eva12562-bib-0071] Taylor, J. W. , Hann‐Sodena, C. , Brancoa, S. , Sylvaina, I. , & Ellison, C. E. (2015). Clonal reproduction in fungi. Proceedings of the National Academy of Sciences, 112, 8901–8908. https://doi.org/10.1073/pnas.1503159112 10.1073/pnas.1503159112 PMC4517272 26195774

[eva12562-bib-0072] Tomsovsky, M. , Tomesova, V. , Palovcikova, D. , Kostovcik, M. , Rohrer, M. , Hanacek, P. , & Jankovsky, L. (2013). The gene flow and mode of reproduction of *Dothistroma septosporum* in the Czech Republic. Plant Pathology, 62, 59–68. https://doi.org/10.1111/ppa.2012.62.issue-1

[eva12562-bib-0073] Vitule, J. R. S. , Freire, C. A. , Vazquez, D. P. , Nunez, M. A. , & Simberloff, D. (2012). Revisiting the potential conservation value of non‐native species. Conservation Biology, 26, 1153–1155. https://doi.org/10.1111/j.1523-1739.2012.01950.x 23083005 10.1111/j.1523-1739.2012.01950.x

[eva12562-bib-0074] Wachowiak, W. , Salmela, M. J. , Ennos, R. A. , Iason, G. , & Cavers, S. (2011). High genetic diversity at the extreme range edge: Nucleotide variation at nuclear loci in Scots pine (*Pinus sylvestris* L.) in Scotland. Heredity, 106, 775–787. https://doi.org/10.1038/hdy.2010.118 20823905 10.1038/hdy.2010.118 PMC3186241

[eva12562-bib-0075] Weir, B. S. (1996). Genetic Data Analysis II. Sunderland, USA: Sinauer Associates Inc.

[eva12562-bib-0076] Weir, B. S. , & Cockerham, C. C. (1984). Estimating *F*‐statistics for the analysis of population structure. Evolution, 38, 1358–1370. doi: 10.1111/j.1558-5646.1984.tb05657.x. 28563791 10.1111/j.1558-5646.1984.tb05657.x

[eva12562-bib-0077] Welsh, C. , Lewis, K. , & Woods, A. (2009). The outbreak history of *Dothistroma* needle blight: An emerging forest disease in northwestern British Columbia, Canada. Canadian Journal of Forest Research, 39, 2505–2519. https://doi.org/10.1139/X09-159

[eva12562-bib-0078] Wingfield, M. J. , Brockerhoff, E. G. , Wingfield, B. D. , & Slippers, B. (2015). Planted forest health: The need for a global strategy. Science, 349, 832–836. https://doi.org/10.1126/science.aac6674 26293956 10.1126/science.aac6674

