# Supplementary material for: Planting exotic relatives has increased the threat posed by Dothistroma septosporum to the Caledonian pine populations of Scotland
Source: Evol Appl. 2017 Nov 10;11(3):350–63. doi: 10.1111/eva.12562 (PMC5881121; doi:10.1111/eva.12562)

**Supplementary Materials for Piotrowska *et al.***

Table S1. The range of allele sizes, number of alleles and allelic richness at 11 microsatellite loci (Barnes *et al.*, 2008) in Scottish populations of *Dothistroma septosporum.*

| Locus | Allele range (bp)^a^ | Number of alleles across all three races | Allelic richness in LPR^b^ | Allelic richness in SR^b^ | Allelic richness in NPR^b^ |
| --- | --- | --- | --- | --- | --- |
| E | 231-278 | 6 | 2.000 | 4.98 | 1.907 |
| F | 192-208 | 4 | 1.000 | 2.000 | 2.686 |
| G | 195-205 | 4 | 1.000 | 3.000 | 2.230 |
| I | 319-328 | 4 | 2.000 | 2.957 | 2.680 |
| J | 203-223 | 7 | 2.000 | 5.518 | 2.773 |
| K | 355-438 | 8 | 1.000 | 7.283 | 2.762 |
| L | 339-459 | 14 | 2.000 | 8.215 | 5.401 |
| M | 233-579 | 37 | 2.000 | 16.38 | 19.85 |
| O | 215-224 | 3 | 1.000 | 1.998 | 1.000 |
| DS1 | 165-175 | 5 | 2.000 | 2.985 | 3.731 |
| DS2 | 388-418 | 6 | 1.000 | 5.120 | 1.543 |

^a^ all allele sizes include M13 tail length

^b^ based on min. sample size of 37 individuals.

Table S2. Pairwise genetic differentiation (*θ_st_*) between SR populations of *Dothistroma septosporum*.

|  | Torrs Warren | Tentsmuir | Culbin Forest | Southern Scottish Nursery |
| --- | --- | --- | --- | --- |
| Caledonian pinewoods | 0.1466 | 0.1134 | 0.0954 | 0.1293 |
| Torrs Warren |  | 0.0613 | 0.0722 | 0.1091 |
| Tentsmuir |  |  | -0.0055 | -0.0085 |
| Culbin Forest |  |  |  | 0.0559 |

Table S3. Pairwise genetic differentiation (*θ_st_*) between NPR populations of *Dothistroma* *septosporum*.

|  | Inshriach Forest | Glen Garry | Dundreggan | Glen Affric | Beinn Eighe | Glen Tanar | Torrs Warren | Tentsmuir | Culbin Forest | Northern Scottish Nursery |
| --- | --- | --- | --- | --- | --- | --- | --- | --- | --- | --- |
| Glen Einig | 0.023 | 0.074 | 0.053 | 0.017 | 0.079 | 0.035 | 0.708 | 0.139 | 0.018 | 0.011 |
| Inshriach Forest |  | 0.087 | 0.067 | 0.038 | 0.060 | -0.017 | 0.623 | 0.115 | 0.028 | 0.009 |
| Glen Garry |  |  | 0.126 | 0.056 | 0.129 | 0.070 | 0.585 | 0.141 | 0.084 | 0.051 |
| Dundreggan |  |  |  | 0.161 | 0.127 | 0.112 | 0.801 | 0.218 | 0.025 | 0.035 |
| Glen Affric |  |  |  |  | 0.064 | 0.020 | 0.638 | 0.127 | 0.025 | 0.027 |
| Beinn Eighe |  |  |  |  |  | 0.108 | 0.681 | 0.191 | 0.055 | 0.055 |
| Glen Tanar |  |  |  |  |  |  | 0.554 | 0.099 | 0.044 | 0.005 |
| Torrs Warren |  |  |  |  |  |  |  | 0.598 | 0.709 | 0.603 |
| Tentsmuir |  |  |  |  |  |  |  |  | 0.158 | 0.077 |
| Culbin Forest |  |  |  |  |  |  |  |  |  | -0.015 |

Table S4. Analysis of variance of growth rate and exudate production for three races of *Dothistroma septosporum* grown at three temperatures. **P*<0.05, ***P*<0.01, ****P*<0.001.

**Source of Variation df MS Growth rate MS Exudate production**

Temperature 2 487.83* 15.03**

Incubator (Temp) 3 30.83*** 0.48

Race 2 475.07** 9.73**

Population (Race) 6 23.30 0.82

Isolate (Race Pop) 36 20.13*** 1.32***

Temp x Race 4 111.09*** 2.73**

Temp x Pop (Race) 12 8.35* 0.49

Temp x Iso (Race Pop) 72 4.37** 0.46*

Error 127 2.52 0.30

Figure S1. Exudate production scoring scale for *Dothistroma septosporum*


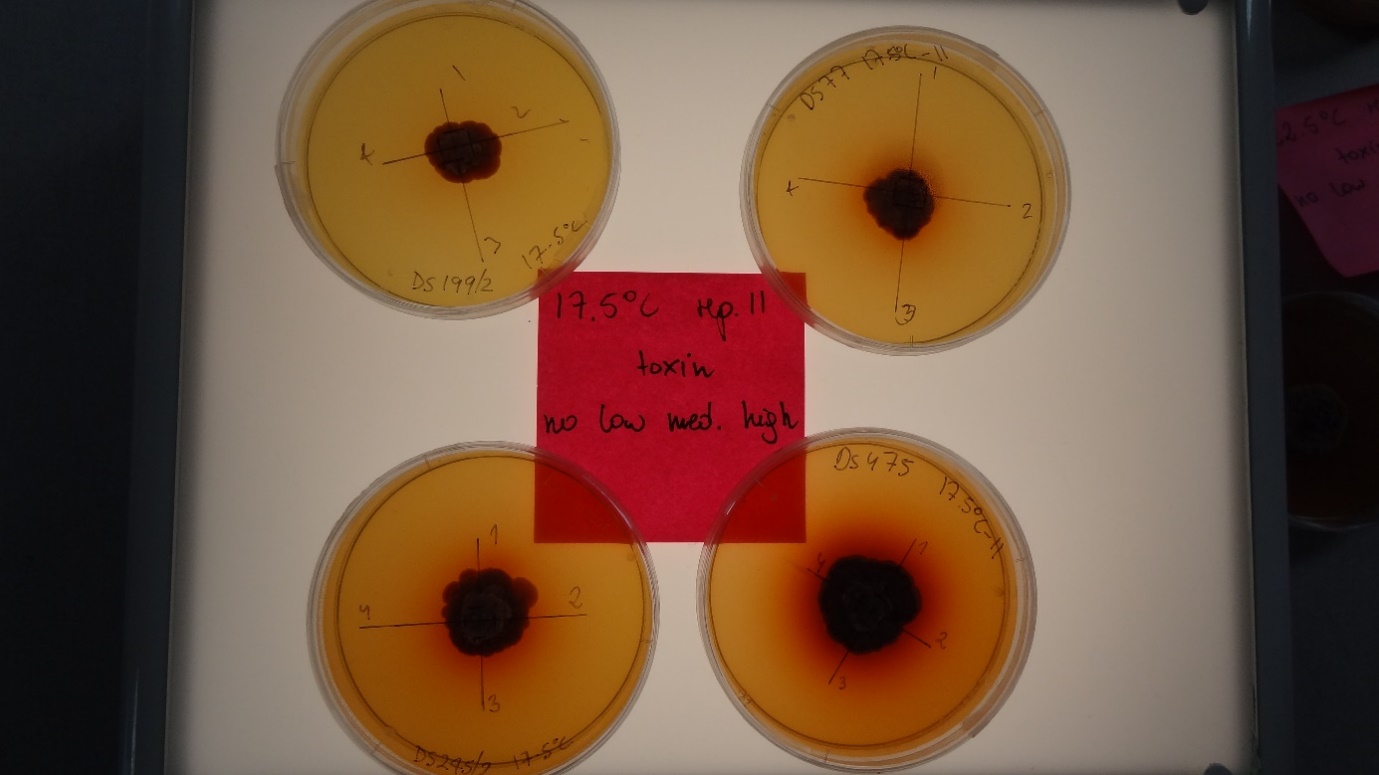

Supplement: Supplementary file 1 [file EVA-11-350-s001.docx]
